# Renal Risk Awareness and Use Patterns of NSAIDs and Antibiotics in Primary Care Patients from North-Eastern Romania

**DOI:** 10.3390/medicina62030594

**Published:** 2026-03-21

**Authors:** Eric Oliviu Cosovanu, Maria Bogdan, Elena Adorata Coman, Cezar Ilie Foia, Cosmin Gabriel Tartau, Elena Teona Cosovanu, Antoneta Dacia Petroaie, Liliana Lacramioara Pavel, Ana-Maria Pelin, Liliana Mititelu Tartau

**Affiliations:** 1 Grigore T. Popa University of Medicine and Pharmacy Iasi, 700115 Iasi, Romania; cosovanu_eric-oliviu@d.umfiasi.ro (E.O.C.); elena.coman@umfiasi.ro (E.A.C.); cezar030699@gmail.com (C.I.F.); cosmin.tartau@gmail.com (C.G.T.); cosovanu_elena-teona@d.umfiasi.ro (E.T.C.); antoneta.petroaie@umfiasi.ro (A.D.P.); lylytartau@yahoo.com (L.M.T.); 2 Doctoral School of Medicine and Pharmacy, Grigore T. Popa University of Medicine and Pharmacy Iasi, 700115 Iasi, Romania; 3 Department of Pharmacology, Faculty of Pharmacy, University of Medicine and Pharmacy, 200349 Craiova, Romania; 4 Medical Department, Faculty of Medicine and Pharmacy, Dunarea de Jos University, 800010 Galati, Romania; doctorpavel2012@yahoo.com; 5 Department of Morphological and Functional Sciences, Faculty of Medicine and Pharmacy, “Dunarea de Jos” University, 800010 Galati, Romania; anapelin@gmail.com

**Keywords:** NSAIDs, antibiotics, self-medication, urban–rural differences, renal risk perception, primary care

## Abstract

*Background and Objectives*: Self-medication and inappropriate use of non-steroidal anti-inflammatory drugs (NSAIDs) and antibiotics are major public health concerns, particularly in settings with variable access to healthcare. Understanding patterns of medication use and renal risk perception can inform targeted interventions. This study examined NSAID and antibiotic use, self-medication behaviors, and renal risk awareness among Romanian primary care patients, with attention to urban–rural differences. *Materials and Methods*: A cross-sectional survey was conducted among 201 primary care patients (101 rural, 100 urban). Data on NSAID and antibiotic use, self-medication practices, sources of recommendation, and renal risk perception were collected using a study-specific questionnaire. Multivariable logistic regression was applied to identify predictors of frequent NSAID use, inappropriate antibiotic use, self-medication frequency, and high perceived renal risk. *Results*: NSAID use was nearly universal (95%), with frequent use strongly associated with non-professional recommendations. Antibiotic misuse was more common in rural participants and largely driven by informal acquisition. Self-medication patterns differed by residence: rural participants reported system- or access-related reasons and reliance on non-professional sources, while urban participants engaged in frequent, convenience-driven self-medication. Although most participants were aware of potential renal harm, this did not consistently lead to safer behaviors. Higher educational level and trust in healthcare professionals predicted increased perceived renal risk, whereas rural residence was associated with lower risk perception. *Conclusions*: Medication misuse is influenced more by recommendation sources, access barriers, and trusted information pathways than by knowledge alone. Interventions should focus on improving professional guidance, addressing informal recommendation networks, and tailoring strategies to urban–rural contexts.

## 1. Introduction

Non-steroidal anti-inflammatory drugs (NSAIDs) and antibiotics represent two of the most frequently used classes of medications worldwide, particularly in primary care and community settings [1]. Their widespread availability, including over-the-counter (OTC) access in many regions, combined with a general perception of safety, has facilitated extensive self-medication practices [2,3].

Nevertheless, inappropriate and unsupervised use of these agents constitutes a major public health concern, given their well-documented potential for renal injury [4,5] and, in the case of antibiotics, their substantial contribution to the development and spread of antimicrobial resistance [6,7].

Self-medication with antibiotics and NSAIDs is common worldwide, driven by the desire for rapid symptom relief, and occurs in both high- and low-/middle-income countries. While perceived as convenient and cost-effective, it poses serious health risks, including nephrotoxicity and contribution to antimicrobial resistance, with up to 50% of antibiotic use considered unnecessary [8,9,10]. Global prevalence of antibiotic self-medication averages 27.7%, with wide regional variation, and national surveys report high rates in countries like Thailand, Jordan, and Brazil [3,11,12,13,14]. Typical misuse behaviors include obtaining drugs without a prescription, using leftovers, altering doses, or stopping treatment early, weak regulation, limited healthcare, and poor patient knowledge [12,13,14,15,16]. These practices increase the risk of gastrointestinal, cardiovascular, and renal complications from NSAIDs and drive antimicrobial resistance from inappropriate antibiotic use [1,17].

Inappropriate use of NSAIDs and antibiotics imposes significant public health and economic burdens. Excessive NSAID use is a largely preventable cause of hospitalizations from acute kidney injury, gastrointestinal bleeding, and cardiovascular events, especially among older adults and children [18]. Antibiotic misuse drives antimicrobial resistance, contributing to an estimated 4.95 million deaths annually and longer, more severe hospitalizations compared with susceptible infections [19,20]. Economic modeling studies suggest that the introduction of new antimicrobials combined with effective stewardship programs could reduce antimicrobial resistance by approximately 9%, gain over 150,000 quality-adjusted life years, and result in savings of several billion US dollars (USD) in healthcare-related costs over a ten-year period [21].

In Romania, self-medication is a significant public health issue, with up to 30% of adults using NSAIDs and 17% using antibiotics without a prescription [22]. Although antibiotics are legally classified as prescription-only medicines in Romania, in accordance with European Union regulations, real-world access has historically varied. In the past, antibiotics were sometimes dispensed without a medical prescription in community pharmacies, only on the recommendation of a pharmacist. In recent years, legislative measures and regulatory controls have been strengthened to limit non-prescription sales and promote responsible antimicrobial use. Nevertheless, previous national studies have reported persistent patterns of self-medication and informal access, particularly in rural areas where access to primary care services may be limited. These findings highlight the discrepancy that can exist between formal regulatory frameworks and everyday practice.

Misconceptions, such as viewing antibiotics as quick remedies for respiratory symptoms and NSAIDs as inherently safe, alongside limited healthcare access, especially in rural areas, drive these practices, with community pharmacies as the main source [23,24]. High-risk groups include children, with 70% of parents reporting self-medication, and adults, with lifetime antibiotic self-medication rates up to 72% [25,26]. NSAIDs and penicillin-based antibiotics are the most commonly misused drugs, while older adults face additional risk from prolonged NSAID prescribing [12,26,27]. Although antibiotics are intended to be used under medical supervision, inappropriate use and premature discontinuation occur in real-world settings. Such practices include initiating antibiotic therapy without a prescription using leftover antibiotics from previous treatments, sharing antibiotics among family members, modifying doses without medical advice, or discontinuing treatment early once symptoms improve. These behaviors are particularly prevalent in settings with limited access to healthcare services.

These patterns mirror broader Eastern European trends, highlighting the need for strengthened pharmacy stewardship and public education to promote safe, rational medication use [28,29,30].

Despite extensive international evidence on the misuse of NSAID and antibiotics and their associated renal and public health risks, significant knowledge gaps remain in Romania. Most national studies have examined isolated aspects of medication use, such as prevalence or prescribing patterns, without integrating patient-reported behaviors, awareness of renal risks, and symptom profiles in primary care. As a result, the true community-level burden of medication-related renal harm is not well defined.

Addressing these gaps is critical for informing preventive strategies that reduce inappropriate medication use, enhance awareness of renal risks, and integrate renal safety into routine primary care. By examining both rural and urban populations within a primary care framework, this study aims to generate evidence directly relevant to community medicine and public health decision-making in Romania and similar Eastern European contexts.

## 2. Materials and Methods

### 2.1. Study Design and Setting

A cross-sectional, questionnaire-based study was conducted between July and December 2025; patients attending urban and rural primary care practices in North-Eastern Romania were invited to participate in the study. The study was designed to explore patterns of NSAID and antibiotic use, self-medication behaviors, healthcare-seeking attitudes, and awareness of potential renal risks, with particular emphasis on urban–rural differences. The sample was consecutive, and no stratification was applied to achieve a specific sex distribution; the observed near-equal ratio of males and females occurred naturally. All patients aged 6 years and older were eligible. For participants under 18 years of age, the survey was completed by a parent or legal guardian. This approach allowed us to capture patterns of NSAID and antibiotic use across the full range of age groups attending primary care.

### 2.2. Survey Development

Data were collected using a structured, self-administered questionnaire developed specifically for this study (Supplementary File S1). The questionnaire was designed based on a review of the literature and reviewed by three physicians with expertise in primary care and clinical pharmacology, who evaluated the clarity, relevance, and comprehensiveness of all survey items prior to data collection. Face validity was assessed through pilot testing prior to data collection. The questionnaire captured information across several domains, including sociodemographic characteristics, patterns of NSAID and antibiotic use (frequency and source of recommendation), self-medication practices, healthcare-seeking behaviors, and perceptions of renal risks associated with medication use.

Prior to data collection, the questionnaire was pilot-tested on 15 participants representing the target population, including adults and parents of younger participants. The pilot testing was conducted by the study team under the supervision of the principal investigator and a primary care physician. The purpose was to assess clarity, comprehension, and completion time. Feedback from the pilot indicated that a few questions, particularly those on NSAID and antibiotic use and on health-seeking behavior, required minor rewording for clarity. These adjustments were incorporated into the final survey; no changes were made to the overall structure or response options. Definitions of variables (sex, pupil/student, age inclusion) were clearly specified to ensure accurate categorization of participants.

In the questionnaire, sex was collected as a biological variable, replacing the previous wording “gender” to ensure accuracy. Regarding educational status, the variable “Pupil” refers to individuals attending primary or secondary school, whereas “Student” refers to individuals enrolled in higher education (college or university). In the questionnaire, examples of commonly used analgesics were provided to facilitate participant understanding. These included ibuprofen and diclofenac (non-steroidal anti-inflammatory drugs, NSAIDs) and paracetamol (acetaminophen), which is not classified as an NSAID but was included as a commonly used analgesic for clarity. For the purpose of statistical analyses presented in this study, only true NSAIDs (e.g., ibuprofen, diclofenac) were considered in NSAID-specific analyses.

### 2.3. Variable Definition and Outcomes

For analytical purposes, NSAID use and antibiotic use were coded as binary variables (yes/no). NSAID use was defined as the use of non-steroidal anti-inflammatory drugs such as ibuprofen or diclofenac, explicitly excluding paracetamol from this category. Frequency of NSAID use was dichotomized into frequent versus rare use and was defined a priori as a primary outcome. Inappropriate antibiotic use was defined as incorrect treatment practices, including non-adherence to prescribed regimens (e.g., premature discontinuation or dose modification). Frequency of self-medication was similarly dichotomized into frequent versus rare use. Sociodemographic variables, including educational level, sex, occupational status, and area of residence (urban vs. rural), were categorized using predefined binary classifications to facilitate regression modeling. In the multivariable logistic regression model, Occupation was included as a categorical variable, with “Employed” as the reference category. Odds ratios for NSAID or antibiotic use were estimated for each category relative to employed participants: Pupils, Students, Unemployed, Homemakers, Retired, and Other. For example, an odds ratio greater than 1 indicates higher odds of the outcome compared to employed participants, while an odds ratio less than 1 indicates lower odds. Definitions of occupation categories are based on survey responses as follows: Pupil (primary/secondary school), Student (higher education), Unemployed, Employed, Homemaker, Retired, Other (specified by participant). For descriptive analyses, denominators were defined according to the number of respondents to each specific item. Conditional questions (e.g., adverse effects, recommendation source) were analyzed only among relevant respondents. For multivariable logistic regression analyses, binary variables were coded as 1 for the exposure category and 0 for the reference category. Specifically, area of residence was coded as urban = 1 and rural = 0 (reference category). Educational level was coded as high = 1 and low = 0. Occupational status was coded as active = 1 and non-active = 0. Recommendation source variables were coded as non-professional = 1 and professional = 0 (reference).

### 2.4. Statistical Analysis

Categorical variables were summarized using frequencies and percentages. Comparisons between urban and rural populations were performed using the Chi-square test or Fisher’s exact test, as appropriate. Multivariable logistic regression analyses were conducted to identify independent predictors of frequent NSAID use, inappropriate antibiotic use, frequent self-medication, and high perceived renal risk. Results were reported as adjusted odds ratios (aORs) with corresponding 95% confidence intervals (CIs). Where sufficient outcome events were available, stratified logistic regression analyses were performed according to area of residence to explore potential effect modification by urban or rural setting. Given the presence of sparse data and potential quasi-complete separation in the logistic regression model for inappropriate antibiotic use, penalized maximum likelihood logistic regression (Firth method) was additionally performed to obtain bias-reduced and more stable estimates. All statistical analyses were conducted using EasyMedStat software Version 3.36 [31], and statistical significance was defined as a two-sided *p*-value < 0.05.

### 2.5. Ethical Aspects

The study was conducted in accordance with international ethical standards for research involving human participants. Ethical approval (Certificate No. 553/06.03.2025) was obtained from the relevant institutional review board prior to initiation, and all participants provided informed consent prior to enrollment. Data collection and handling were performed in full compliance with confidentiality requirements and applicable ethical guidelines, ensuring respect for participant autonomy, privacy, and safety throughout the study [32]. All procedures adhered to the principles outlined in the Declaration of Helsinki [33] and other relevant international research norms.

## 3. Results

### 3.1. Participants’ Characteristics

A total of 201 participants were enrolled in the study, with an approximately equal distribution between rural (n = 101, 50.3%) and urban (n = 100, 49.7%) settings. The study cohort comprised both male and female participants, with females representing 53.2% (n = 107) and males 46.8% (n = 94) of the sample.

The majority of participants were adults, with 47.8% aged 20–39 years, 29.4% aged 40–59 years, and 18.9% aged ≥60 years. A minority of participants were younger than 20 years (4%). Regarding educational attainment, participants were almost evenly distributed between low (52.2%) and high (47.8%) education levels. In terms of employment status, 36.8% of the cohort were actively employed, whereas 63.2% were classified as non-active.

Concerning medical history, 29.9% of participants reported a pre-existing chronic condition, including renal or other chronic diseases, while 70.1% reported no chronic diagnoses. Reported chronic morbidity mainly encompassed cardiometabolic conditions, including hypertension and diabetes, as well as chronic kidney disease and persistent respiratory or allergic disorders. Detailed characteristics of the study population are summarized in Table 1.

### 3.2. Descriptive Patterns of NSAID and Antibiotics Use, Self-Medication Behaviors and Healthcare Attitudes

An overview of NSAID and antibiotic use, self-medication practices, and healthcare-related attitudes is presented in Table 2. NSAID use was reported by 95.36% of participants at least once in their lifetime. Frequent NSAID use was noted in 54 participants (27%), whereas the remainder reported rare or occasional use. NSAIDs were obtained following a healthcare professional’s recommendation by 100 respondents (49.75%), while a comparable proportion reported non-professional or self-initiated use (n = 101, 50.25%). Adverse effects associated with NSAID use were reported by 16 participants, with urticaria and somnolence being the most commonly described. This item was completed only by respondents who provided information regarding adverse effects, resulting in a smaller denominator than the total number of NSAID users.

Antibiotic use within the preceding year was reported by 107 participants (60.8%). Among these, 46 cases involved non-professional recommendation or self-medication, while 87 participants received antibiotics following professional medical advice. Participants were allowed to report more than one source of recommendation, which explains why the total number of responses exceeds the number of antibiotic users.

Inappropriate antibiotic use was reported by 53 respondents (26.37%), whereas correct use was reported by the majority (n = 148, 73.63%). Inappropriate antibiotic use was defined as any antibiotic-related behavior deviating from medical recommendations, including initiation of antibiotic therapy without a prescription, use of leftover antibiotics from previous treatments, modification of prescribed doses or duration without medical advice, premature discontinuation of treatment once symptoms improved, or use of antibiotics for non-bacterial or non-indicated conditions. Antibiotic-related adverse effects were infrequently reported (n = 16, 12.12%), with diarrhea being the most commonly mentioned symptom.

Self-medication behaviors were prevalent across the study population. Frequent self-medication was reported by 126 participants (63%), while 74 participants reported occasional self-medication. Non-professional sources of information were more frequently consulted during self-medication (n = 112, 55.72%) compared with professional sources (n = 89, 44.28%). Medications obtained without a prescription were sourced almost equally from non-professional (n = 103, 51.24%) and professional settings (n = 98, 48.76%). A total of 95 participants (66.9%) reported experiencing adverse effects related to self-medication. When experiencing pain, self-medication was the initial response for the majority of respondents (n = 184, 91.54%), with a similar pattern observed for fever (n = 147, 73.13%).

Regarding healthcare-related attitudes and renal risk perception, most participants indicated willingness to undergo urine testing if recommended (n = 188, 93.53%), whereas avoidance of medical advice or consultation was commonly reported (n = 190, 95.48%). A majority recognized that OTC medications could adversely affect renal function (n = 142, 87.12%), although only 35 participants (32.11%) reported having been advised to undergo blood tests to monitor renal function before or after NSAID or antibiotic use. Trust in healthcare professionals as the most reliable source of information on medication-related risks was expressed by 175 respondents (87.06%).

With respect to medical history, hypertension and diabetes were the most frequently reported chronic conditions, followed by allergic rhinitis and atopic dermatitis. Among participants reporting urinary or renal-related symptoms, the most commonly described manifestations included back pain, fatigue, and changes in urinary appearance. Changes in urinary appearance referred to self-reported abnormalities such as dark-colored or cloudy urine, foamy urine, visible blood, or unusual odor.

### 3.3. Urban–Rural Comparisons of NSAID and Antibiotic Use Patterns, Self-Medication Behaviors and Healthcare Attitudes

Urban–rural comparisons are summarized in Table 3. Associations between place of residence and medication-related behaviors or healthcare attitudes were evaluated using the Chi-square test or Fisher’s exact test, as appropriate.

NSAID use was highly prevalent in both rural and urban participants, with no significant differences in overall use, frequency, or NSAID-related adverse effects. However, rural participants were significantly more likely to report non-professional recommendations for NSAID use (*p* < 0.001).

Overall, antibiotic use and antibiotic-related adverse effects did not differ significantly between groups. In contrast, inappropriate antibiotic use was significantly more common among rural participants compared with urban participants (36.6% vs. 16%, *p* = 0.002).

Self-medication behaviors differed markedly between groups. Frequent self-medication was significantly more common among urban participants (*p* < 0.001), whereas rural participants more frequently cited access- or system-related reasons for self-medication and relied more on non-professional information sources (both *p* < 0.001). Perceived harmful effects associated with self-medication were reported more frequently by urban respondents (*p* < 0.001).

Regarding healthcare-seeking behaviors, urban participants were more likely to report non-professional first responses to pain (*p* = 0.009), while no significant differences were observed for responses to febrile or infectious symptoms. Willingness to undergo urine testing and avoidance of medical consultation were similar between the two groups.

Finally, urban participants more frequently acknowledged the potential renal risks of OTC medications (*p* = 0.018), whereas rural participants were more likely to report having undergone renal function testing before or after NSAID or antibiotic use (*p* < 0.001). Trust in healthcare professionals as sources of medication-related information was high and comparable across both groups.

Data are presented as counts (n) and percentages (%). For all variables, only the “Yes” category is reported, representing the presence of the specified behavior, exposure, or perception. Percentages were calculated within each subgroup (urban or rural). Statistical comparisons were performed using the Chi-square test or Fisher’s exact test, as appropriate.

### 3.4. Predictors of Frequent NSAID Use

#### 3.4.1. Global Analysis

Predictors of frequent NSAID use were assessed using multivariable logistic regression in the overall study population. The model included sex, area of residence (urban vs. rural), education level, occupational status, and source of NSAID recommendation. In the adjusted model, non-professional recommendation of NSAIDs was independently associated with a higher likelihood of frequent NSAID use (OR 3.25, 95% CI 1.53–6.93, *p* = 0.002). Conversely, being professionally active was associated with a significantly lower likelihood of frequent NSAID use (OR 0.32, 95% CI 0.14–0.71, *p* = 0.005). Educational level, area of residence, and male sex were not independently associated with frequent NSAID use (*p* > 0.05 for all). Results of the multivariable model are summarized in Table 4.

#### 3.4.2. Stratified Analysis by Area of Residence

To further investigate potential differences by place of residence, stratified multivariable logistic regression analyses were performed. In the rural subgroup, being professionally active was independently associated with a lower likelihood of frequent NSAID use (OR = 0.17, 95% CI 0.04–0.63, *p* = 0.008). In contrast, source of NSAID recommendation, educational level, and sex were not independently associated with frequent NSAID use in this subgroup (*p* > 0.05 for all). Results of the rural stratified model are presented in Table 5. In the urban subgroup, stratified multivariable analysis did not identify any independent predictors of frequent NSAID use. Due to limited variability of the outcome and an insufficient number of events relative to the number of explanatory variables, a stable multivariable model could not be constructed for urban participants.

### 3.5. Predictors of Inappropriate Antibiotic Use

#### 3.5.1. Global Analysis

Predictors of inappropriate antibiotic use were assessed using multivariable logistic regression in the overall study population. The model included the source of antibiotic recommendation, frequency of self-medication, educational level, occupational status, and presence of a known chronic disease. In the standard multivariable logistic regression model, obtaining antibiotics without professional medical recommendation was strongly associated with inappropriate antibiotic use (OR = 196.71, 95% CI 34.24–1130.1, *p* < 0.0001). However, due to sparse data and quasi-complete separation, this estimate was likely inflated. To address this issue, a penalized logistic regression using the Firth method was performed. The association remained statistically significant, although the effect size was attenuated (Firth-adjusted OR = 42.58, 95% CI 12.14–176.32, *p* < 0.001), confirming the robustness of the finding while providing a more conservative and bias-reduced estimate. None of the other variables included in the model were independently associated with inappropriate antibiotic use (all *p* > 0.05). Results of the global multivariable analysis are summarized in Table 6.

#### 3.5.2. Stratified Analysis by Area of Residence

In the rural subgroup, multivariable logistic regression analysis did not identify any independent predictors of inappropriate antibiotic use. Occupational status, presence of a known chronic disease, and frequency of self-medication were not significantly associated with inappropriate antibiotic use in this subgroup (all *p* > 0.05). Results of the rural stratified analysis are presented in Table 7. In the urban subgroup, a multivariable regression model could not be reliably fitted due to an insufficient number of inappropriate antibiotic treatment events, resulting in sparse data and unstable estimates. Consequently, no independent predictors could be identified for urban participants.

### 3.6. Medication-Related Behaviors and Renal Risk Awareness Among NSAID and Antibiotics Users

#### 3.6.1. Predictors of Frequent Self-Medication

Predictors of frequent self-medication were assessed using multivariable logistic regression in the overall study population. The model included area of residence, educational level, occupational status, presence of a known chronic disease, and source of NSAID and antibiotic recommendations. Multivariable logistic regression was performed to identify factors associated with NSAID and antibiotic use. Occupation was included as a categorical variable, with “Employed” serving as the reference category. Odds ratios (ORs) and 95% confidence intervals (CIs) were estimated for each category relative to employed participants. The occupation categories, as reported in the survey, were defined as follows: Pupil (primary or secondary school), Student (higher education), Unemployed, Homemaker, Retired, and Other (specified by participant). An OR greater than 1 indicates higher odds of the outcome compared to employed participants, whereas an OR less than 1 indicates lower odds. For example, students had an OR of X.X (95% CI: X.X–X.X) for frequent NSAID use, indicating higher/lower odds relative to employed participants.

In the adjusted analysis, non-professional NSAID recommendation was independently associated with a higher frequency of self-medication (OR = 3.09, 95% CI 1.14–8.33, *p* = 0.026). Area of residence was also a strong predictor. Urban residence (coded as 1, with rural as reference) was associated with significantly lower odds of frequent self-medication (OR = 0.04, 95% CI 0.01–0.21, *p* < 0.001), indicating that rural participants had a substantially higher likelihood of frequent self-medication compared with urban participants.

Educational level, occupational status, presence of chronic disease, and non-professional antibiotic recommendation were not independently associated with frequent self-medication in the multivariable model (all *p* > 0.05). Results of the analysis are presented in Table 8.

#### 3.6.2. Predictors of Perceived Renal Risk Related to Medication Use

An initial multivariable logistic regression model was constructed to assess predictors of high perceived renal risk associated with NSAID and antibiotic use. The model included area of residence, frequency of self-medication, educational level, and source of NSAID and antibiotic recommendations. In this model, none of the variables were independently associated with high perceived renal risk (all *p* > 0.05). Although some variables showed non-significant trends, no statistically significant associations were identified. Results of the model are presented in Table 9.

Given the conceptual relevance of information sources and health literacy, a subsequent multivariable model was constructed focusing on educational level and trust in information sources regarding medication-related risks. In this adjusted model, higher educational level was independently associated with increased perceived renal risk (OR = 3.46, 95% CI 1.04–11.49, *p* = 0.043). Trust in healthcare professionals as the most reliable source of information was also strongly associated with higher perceived renal risk (OR = 12.74, 95% CI 3.01–53.91, *p* = 0.001). Conversely, rural residence was independently associated with lower perceived renal risk compared with urban residence (OR = 0.14, 95% CI 0.04–0.53, *p* = 0.003). The primary source of OTC medication information was not independently associated with perceived renal risk. Results of the analysis are summarized in Table 10.

Overall, frequent self-medication was primarily associated with non-professional NSAID recommendation and rural residence, whereas perceived renal risk was influenced more by educational level and trust in healthcare professionals than by self-medication behaviors alone.

## 4. Discussions

In Romania, the risk of renal injury from NSAID and antibiotic misuse varies across populations. Rural residents and older adults are particularly vulnerable due to limited healthcare access, reliance on pharmacies, and high rates of inappropriate NSAID use [27,34,35]. Lower education and income, as well as younger adults influenced by prior prescriptions or family advice, further increase self-medication rates [36]. Economic and structural factors, including low reimbursement and high co-payments, promote OTC or leftover drug use [37]. These patterns, shaped by age, location, socioeconomic status, and healthcare access, reflect broader Eastern European trends and underscore the need for targeted, equity-focused interventions.

This study provides a comprehensive analysis of NSAID and antibiotic use, self-medication behaviors, and renal risk awareness among primary care patients in Romania, highlighting key differences between urban and rural populations. The findings underscore the multifactorial drivers of medication practices in community settings, combining availability, social influences, access to care, and health literacy.

NSAID consumption was highly prevalent across both urban and rural participants, reflecting their widespread availability and common perception as low-risk, OTC medications. While overall use and frequency did not differ significantly between areas, rural participants were more likely to rely on non-professional recommendations, indicating that informal advice networks play a critical role in repeated NSAID exposure. Multivariable logistic regression confirmed that non-professional recommendation was the strongest predictor of frequent NSAID use, independent of education, occupational status, or residence. This finding highlights the importance of informal guidance, such as advice from family, friends, or pharmacists, in shaping repeated NSAID consumption, suggesting that interventions targeting safe NSAID use should address these community-level influences. Interestingly, professional activity was associated with reduced likelihood of frequent NSAID use, potentially reflecting greater health awareness, structured routines, or increased access to healthcare guidance among employed participants.

Antibiotic use was also common, with a significant proportion of participants reporting inappropriate use, particularly in rural areas. This aligns with prior evidence linking limited healthcare access, reduced prescription oversight, and informal acquisition of antibiotics to misuse. The very strong association between non-professional recommendation and inappropriate antibiotic use (Firth-adjusted OR ≈ 43) emphasizes that informal channels remain the dominant driver of misuse, even after applying bias-reduced penalized regression to account for sparse data. Educational level, occupational status, and chronic disease presence were not independently associated, suggesting that accessibility and source of advice outweigh individual demographic or health factors in predicting inappropriate use. These findings underscore the need for targeted educational campaigns and stricter regulatory enforcement to curb informal antibiotic use, particularly in rural communities.

Distinct urban–rural patterns emerged in self-medication practices. Rural participants more frequently reported access- or system-related reasons for self-medication, alongside greater reliance on non-professional information sources and medication acquisition pathways. In contrast, urban participants demonstrated a higher overall frequency of self-medication, likely reflecting convenience, time constraints, or perceived efficiency rather than structural barriers. Multivariable analysis confirmed that rural residence and non-professional NSAID recommendation were independent predictors of frequent self-medication, whereas antibiotic recommendation did not independently influence these behaviors. These results suggest that self-medication is driven by different mechanisms across settings: systemic barriers and informal networks in rural areas, and convenience-driven behaviors in urban areas. Interventions to reduce self-medication must therefore be tailored to these context-specific determinants.

Renal injury is a significant complication associated with inappropriate use of NSAIDs and antibiotics. NSAIDs induce nephrotoxicity primarily through cyclooxygenase inhibition, which reduces prostaglandin synthesis, impairs intrarenal hemodynamics, and decreases glomerular filtration, potentially resulting in acute kidney injury. Chronic NSAID use may further cause immune-mediated damage, including interstitial nephritis, papillary necrosis, and fibrosis [38,39,40]. The nephrotoxic effects of antibiotics vary by class: aminoglycosides cause dose-dependent tubular necrosis, vancomycin leads to both tubular and immune-mediated injury, and β-lactams may provoke hypersensitivity-related interstitial nephritis [41]. Co-exposure to NSAIDs and antibiotics can have synergistic nephrotoxic effects, particularly in older adults, patients with chronic kidney disease, and individuals on multiple medications, underscoring the importance of rational drug use, patient education, and careful renal monitoring to prevent kidney damage [27,39,41]. Histopathologically, NSAID- and antibiotic-induced renal toxicity produces distinct alterations in both tubular and interstitial compartments [5,41].

Studies examining community antibiotic use reveal pronounced urban–rural disparities in both knowledge and behavior. In Bosnia and Herzegovina, urban participants exhibited greater understanding of appropriate antibiotic use, while self-medication and irregular consumption were more prevalent in rural communities [42]. Likewise, a Romanian KAP study reported generally satisfactory knowledge of antibiotic indications and associated risks, yet highlighted persistent discrepancies between knowledge and actual practices [43]. Collectively, these findings indicate that knowledge alone is insufficient to curb inappropriate antibiotic use, particularly in settings marked by urban–rural inequalities [42,43].

Converging evidence suggests that NSAID use remains prevalent among individuals at elevated risk of renal impairment. In Poland, patients with chronic kidney disease (CKD) reported frequent NSAID consumption alongside limited awareness of potential renal adverse effects [44]. Similarly, a study from Egypt found that nearly two-thirds of CKD patients regularly used NSAIDs, often for prolonged periods and frequently in combination with interacting medications [45]. In Sweden, NSAID exposure was also widespread among older adults, including those with reduced kidney function, underscoring age-related susceptibility to NSAID-induced nephrotoxicity [46].

Collectively, these studies indicate that inappropriate medication use patterns and insufficient risk awareness persist for both antibiotics and NSAIDs, thereby contributing to avoidable adverse outcomes, including antimicrobial resistance and renal injury. These findings underscore the need for integrated interventions focusing on medication literacy, patient education, and effective risk communication, particularly across urban and rural populations [41,42,43,44,45,46].

In our study, awareness of potential renal harm associated with OTC medication use was high across the population, yet this knowledge did not consistently translate into safer medication practices. Multivariable analyses revealed that higher educational level and trust in healthcare professionals were independently associated with increased perceived renal risk, while rural residence was linked to lower risk perception. These findings suggest a disconnect between general awareness and behavioral change: even participants who recognize potential risks may continue to engage in high-frequency or inappropriate medication use if structural or social influences dominate their decision-making. The data highlight the importance of not only disseminating knowledge but also promoting practical guidance and reinforcing professional counseling to bridge the gap between awareness and behavior.

The present study indicates that medication misuse in primary care populations is shaped more by the source of recommendations, access to healthcare, and information pathways than by risk awareness alone. Informal advice networks, rural access barriers, and convenience-driven urban behaviors interact to influence NSAID and antibiotic use as well as self-medication patterns. Efforts to improve medication safety should therefore combine regulatory enforcement, community education, and targeted interventions that account for urban–rural disparities. In particular, addressing non-professional recommendation pathways and strengthening trust and access to professional healthcare advice may have the greatest impact on reducing frequent NSAID use, inappropriate antibiotic use, and unsafe self-medication behaviors.

The questionnaire used in this study was developed based on previously published survey instruments assessing medication use, knowledge, attitudes, and behaviours in community and population settings [43,47,48]. Similar cross-sectional questionnaires have been used to evaluate knowledge and attitudes toward antibiotic use and resistance in general populations, incorporating items on self-medication, perceptions, and behaviours relating to antibiotic consumption and awareness of antimicrobial resistance [36,43]. Likewise, studies assessing awareness, knowledge, and attitudes toward NSAID and analgesic use have employed structured questionnaires with domains covering patterns of use, knowledge of risks, and attitudes toward safety and self-medication practices [49]. These instruments demonstrate that sections on health-seeking behaviour and patient perceptions can provide insights into factors that contribute to inappropriate use of medications, even when routine laboratory testing is not clinically required. Including these sections enables analysis of determinants such as consultation behaviour, self-medication practices, and awareness of adverse effects, which are relevant to understanding and improving rational NSAID and antibiotic use.

Several limitations should be considered when interpreting the findings of this study. First, the cross-sectional design precludes any causal inferences; the associations identified between non-professional recommendations, self-medication, and perceived renal risk cannot establish temporality or directionality. Second, the study relied on self-reported data, which may be subject to recall bias, social desirability bias, or misreporting of medication use, frequency, or adverse effects. Third, the questionnaire used to assess medication behaviors, self-medication, and renal risk awareness was specifically developed for this study and has not been formally validated. As a result, measurement error or limited reliability may have affected the accuracy and consistency of the responses. Fourth, the sample, although balanced between urban and rural participants, was limited to primary care attendees in selected regions of Romania, which may reduce generalizability to other populations, regions, or healthcare systems. Finally, certain subgroups, particularly urban participants reporting frequent self-medication or inappropriate antibiotic use, had limited event numbers, resulting in sparse data and unstable estimates in stratified multivariable models.

Despite these limitations, the study provides valuable insights into urban–rural differences in medication use, self-medication behaviors, and renal risk awareness, highlighting the central role of recommendation sources and access-related factors in shaping community medication practices.

## 5. Conclusions

NSAID and antibiotic use are highly prevalent among Romanian primary care patients, with substantial levels of self-medication and inappropriate antibiotic use. Non-professional recommendations emerged as a major driver of frequent NSAID consumption and antibiotic misuse, while rural residence was associated with increased vulnerability to inappropriate medication practices and lower perceived renal risk. Although most participants were aware of potential renal harm from OTC medications, this knowledge did not consistently translate into safer behaviors. These findings highlight the need for targeted interventions in primary care and pharmacy settings that address both access-related barriers and informal recommendation pathways, rather than focusing solely on patient education.

## Figures and Tables

**Table 1:** Detailed characteristics of study participants.

Characteristic	Category	n	%
Age group (years)	<20	8	3.98
	20–39	96	47.76
	40–59	59	29.35
	>59	38	18.91
Sex	Female	107	53.23
	Male	94	46.77
Area of residence	Urban	100	49.75
	Rural	101	50.25
Education level	Low	105	52.24
	High	96	47.76
Occupational status	Non-Active	127	63.18
	Active	74	36.82
Known chronic diagnosis	No	141	70.15
	Yes	60	29.85

Data are presented as counts (n) and corresponding percentages (%). Educational level was categorized as low (comprising primary, secondary, and post-secondary education) and high (including university and postgraduate studies). Occupational status was classified as active (employed participants) or non-active (students, unemployed individuals, retirees, and homemakers). Chronic diagnoses included self-reported physician-diagnosed conditions such as arterial hypertension, diabetes mellitus, chronic kidney disease, cardiovascular disease, and chronic respiratory or allergic conditions.

**Table 2:** Patterns of NSAID and antibiotic use, self-medication behaviors, and healthcare-related attitudes among the study participants.

Variable	Category	n	%	Denominator (n)
NSAID use	Yes	185	95.4	194
	No	9	4.6	194
Frequent NSAID use	Frequent	54	27.0	200
	Rare	146	73.0	200
NSAID recommendation †	Non-professional	101	50.3	201
	Professional	100	49.7	201
NSAID adverse effects †	Yes	16	12.5	128
	No	112	87.5	128
Antibiotic use (past year)	Yes	107	60.8	176
	No	69	39.2	176
Antibiotic recommendation † ‡	Non-professional	46	34.6	133
	Professional	87	65.4	133
Inappropriate antibiotic use	Incorrect	53	26.4	201
	Correct	148	73.6	201
Frequent self-medication	Frequent	126	63.0	200
	Rare	74	37.0	200
Perceived renal risk of OTC medication	Yes	142	87.1	163
	No	21	12.9	163
Trust in healthcare professionals	Yes	175	87.1	201
	No	26	12.9	201

Data are presented as counts (n) and corresponding percentages (%). Percentages were calculated based on the number of respondents for each specific item; consequently, denominators differ across variables due to missing responses. Frequent NSAID use was defined as self-reported use occurring at least once per week or more often during the previous year. Frequent self-medication was defined as regular use of medications without medical advice for common symptoms (e.g., pain, fever, or minor infections), reported as occurring often or very often. For the item “Antibiotic recommendation,” multiple responses were permitted; therefore, the summed frequencies may exceed the total number of participants reporting antibiotic use. For the item “NSAID adverse effects,” the denominator includes only participants who responded to this specific question; therefore, totals may not correspond to the overall number of NSAID users due to missing responses. † Denominator includes only participants who responded to the adverse effects question. ‡ Multiple responses were permitted for antibiotic recommendation.

**Table 3:** Urban–rural comparisons of medication use and healthcare behaviors.

Variable (Yes Category)	Rural n (%)	Urban n (%)	Denominator (Rural/Urban)	*p*-Value
NSAID use	87 (92.6)	98 (98.0)	94/100 †	0.093
Frequent NSAID use	30 (30.0)	24 (24.0)	101/100	0.426
Non-professional NSAID recommendation †	71 (70.3)	30 (29.0)	101/100	<0.001
NSAID adverse effects ‡	7 (12.7)	9 (12.3)	55/73	>0.999
Antibiotic use	52 (65.8)	55 (56.7)	79/97	0.281
Non-professional antibiotic recommendation †§	27 (36.5)	19 (32.2)	74/59	0.740
Inappropriate antibiotic use §	37 (36.6)	16 (16.0)	101/100	0.002
Frequent self-medication	39 (38.6)	87 (87.9)	101/99	<0.001
Access-related reason for self-medication	33 (32.7)	10 (10.0)	101/100	<0.001
Non-professional information source †	55 (54.5)	57 (57.0)	101/100	0.825
Medication obtained without prescription †	68 (67.3)	35 (35.0)	101/100	<0.001
Perceived renal risk of OTC medication	65 (80.3)	77 (93.9)	81/82	0.018
Self-medication as first reaction to pain	87 (86.1)	97 (97.0)	101/100	0.009
Willingness to undergo urine testing	96 (95.0)	92 (92.0)	101/100	0.407
Trust in healthcare professionals	87 (86.1)	88 (88.0)	101/100	0.855

Data are presented as counts (n) and percentages (%). Percentages were calculated within each subgroup (rural or urban), based on the number of respondents to the specific item. Denominators are explicitly indicated for clarity and may differ due to missing responses or conditional question structure. † Multiple responses were permitted. ‡ Denominator includes only respondents who answered the adverse effects question. § Calculated among participants who reported antibiotic use.

**Table 4:** Multivariable analysis of frequent NSAID use.

Variable	Adjusted OR	95%CI	*p*-Value
Non-professional NSAID recommendation	3.25	1.53–6.93	0.002
Occupation (employed vs. non-active)	0.32	0.14–0.71	0.005
Education (high vs. low)	0.86	0.35–2.14	0.744
Area of residence (urban vs. rural)	1.71	0.67–4.39	0.263
Sex (male vs. female)	1.89	0.96–3.71	0.063

Multivariable logistic regression was used to evaluate predictors of frequent NSAID use. Results are presented as adjusted odds ratios (ORs) with corresponding 95% confidence intervals (CIs). Frequent NSAID use: Defined as regular use of NSAIDs, based on questionnaire responses; rare or occasional use was coded separately. Reference categories: rural residence, low education, non-active occupational status, and professional recommendation (unless otherwise specified).

**Table 5:** Rural stratified analysis by area of residence.

Variable	Adjusted OR	95%CI	*p*-Value
Occupation (employed vs. non-active)	0.17	0.04–0.63	0.008
Non-professional NSAID recommendation	1.32	0.51–3.46	0.566
Education (high vs. low)	1.37	0.35–5.37	0.655
Sex (male vs. female)	1.53	0.61–3.85	0.364

Multivariable logistic regression was conducted to identify predictors of frequent NSAID use in the rural subgroup. Results are presented as adjusted odds ratios (ORs) with corresponding 95% confidence intervals (CIs). Frequent NSAID use: Defined as regular use of NSAIDs, based on questionnaire responses; rare or occasional use was coded separately. Reference categories: rural residence, low education, non-active occupational status, and professional recommendation (unless otherwise specified).

**Table 6:** Penalized multivariable analysis of inappropriate antibiotic use (Firth logistic regression).

Variable	Adjusted OR	95%CI	*p*-Value
Non-professional antibiotic recommendation	42.58	12.14–176.32	<0.0001
Education	0.32	0.07–1.44	0.139
Occupation	2.52	0.52–12.30	0.254
Frequent self-medication	2.67	0.56–12.72	0.216
Known chronic disease	3.77	0.71–20.09	0.120

Multivariable logistic regression was used to evaluate predictors of inappropriate antibiotic use in the overall study population. Results are presented as adjusted odds ratios (ORs) with corresponding 95% confidence intervals (CIs). All listed variables were entered simultaneously into the model. Inappropriate antibiotic use: Defined as any self-reported behavior including not completing a prescribed course, using leftover antibiotics, or taking antibiotics without a prescription. Participants reporting any of these behaviors were classified as having inappropriate antibiotic use. Reference categories: rural residence, low education, non-active occupational status, and professional recommendation (unless otherwise specified).

**Table 7:** Residence-stratified analysis.

Variable	Adjusted OR	95%CI	*p*-Value
Occupation	0.76	0.3–1.89	0.555
Known chronic disease	0.76	0.32–1.81	0.536
Frequent self-medication	1.68	0.72–3.89	0.227

Multivariable logistic regression was used to evaluate predictors of inappropriate antibiotic use in the rural subgroup. Results are presented as adjusted odds ratios (ORs) with corresponding 95% confidence intervals (CIs). Due to limited variability and a low number of events, other variables were excluded from the stratified model. Inappropriate antibiotic use: Defined as any self-reported behavior including not completing a prescribed course, using leftover antibiotics, or taking antibiotics without a prescription. Participants reporting any of these behaviors were classified as having inappropriate antibiotic use. Reference categories: rural residence, low education, non-active occupational status, and professional recommendation (unless otherwise specified).

**Table 8:** Frequent self-medication: multivariable predictors.

Variable	Adjusted OR	95%CI	*p*-Value
Non-professional NSAID recommendation	3.09	1.14–8.33	0.026
Area of residence	0.04	0.01–0.21	<0.001
Education	0.41	0.09–1.95	0.265
Known chronic disease	0.72	0.3–1.75	0.466
Occupation	0.74	0.29–1.91	0.531
Non-professional antibiotic recommendation	0.88	0.32–2.39	0.805

Multivariable logistic regression model evaluating predictors of frequent self-medication in the overall study population. Results are presented as adjusted odds ratios with corresponding 95% confidence intervals.

**Table 9:** Perceived renal risk: predictors.

Variable	Adjusted OR	95%CI	*p*-Value
Non-professional antibiotic recommendation	0.34	0.11–1.07	0.065
Area of residence	0.46	0.1–2.22	0.334
Frequent self-medication	0.93	0.28–3.13	0.904
Non-professional NSAID recommendation	1.42	0.42–4.85	0.575
Education	3.55	0.82–15.29	0.089

Initial multivariable logistic regression was performed to evaluate predictors of high perceived renal risk associated with NSAID and antibiotic use.

**Table 10:** Education and trusted sources: predictors of risk perception.

Variable	Adjusted OR	95%CI	*p*-Value
Education	3.46	1.04–11.49	0.043
Trust in healthcare professionals	12.74	3.01–53.91	0.001
Area of residence	0.14	0.04–0.53	0.003
Primary OTC information source	0.57	0.22–1.46	0.241

Multivariable logistic regression was conducted to evaluate educational level and trusted information sources as predictors of perceived renal risk associated with medication use.

## Data Availability

The original contributions presented in this study are included in the article/supplementary material. Further inquiries can be directed to the corresponding author.

## Supplementary Materials

The following supporting information can be downloaded at: https://www.mdpi.com/article/10.3390/medicina62030594/s1.
