# Supplementary material for: Renal Risk Awareness and Use Patterns of NSAIDs and Antibiotics in Primary Care Patients from North-Eastern Romania
Source: Medicina (Kaunas). 2026 Mar 21;62(3):594. doi: 10.3390/medicina62030594 (PMC13027675; doi:10.3390/medicina62030594)
Supplement: Supplementary file 1 [file medicina-62-00594-s001.zip › medicina-4146977-supplementary.pdf]

Questionnaire on the Use of Non-Steroidal Anti-Inflammatory Drugs (NSAIDs) and Antibiotics

Instructions: Please answer the following questions honestly. Your responses are anonymous and confidential. Circle or X one option when applicable.

I. Sociodemographic data

1. What is your age?
- o <20 years
- o 20-39 years
- o 40-59 years
- o >59 years

2. Which is your sex?
- o Female
- o Male
- o Prefer not to answer

3. What is your current occupational status?
- o Pupil
- o Student
- o Unemployed
- o Employed
- o Homemaker
- o Retired
- o Other (please specify):__________

["Pupil" refers to individuals attending primary or secondary school,"Student" refers to individuals enrolled in higher education (college or university)]

4. What is your level of education?
- o Primary/lower secondary
- o Upper secondary
- o Post-secondary
- o University
- o Postgraduate

5. What type of area do you live in?
- o Urban
- o Rural

6. Do you have a known diagnosis of kidney disease or other chronic conditions?
- o Yes, kidney disease
- o Yes, other chronic diseases
- o No

7. Which diseases do you know you suffer from?

II. Use of non-steroidal anti-inflammatory drugs (NSAIDs) and analgesics

8. Have you ever used painkillers (e.g., paracetamol) or non-steroidal anti-inflammatory drugs (NSAIDs) (e.g., ibuprofen, diclofenac)?

- Yes
- No
- I don't know

9. How often do you use painkillers?

- Daily
- Weekly
- Occasionally
- Very rare/never

10. Painkillers are usually recommended by:

- Family physician/specialist
- Pharmacist
- Friends/family
- I take them on my own

11. Have you ever experienced adverse effects after using painkillers?

- Yes
- No
- I don't know/ I don't remember

12. Which adverse effects did you experience?

III. Antibiotic use

13. Have you taken antibiotics in the past year?

- Yes
- No
- I don't remember

14. Who recommended the antibiotic?

- Physician
- Pharmacist
- Friends/family
- I self-medicated

15. When using antibiotics, you usually:

- Finish the full treatment course
- Stop when symptoms disappear
- Adjust the dose on my own
- Combine with other medications without recommendation

16. Have you ever experienced adverse effects after using antibiotics?

- Yes
- No
- I don't know/ I don't remember

17. Which adverse effects did you experience?

IV. Self-medication

18. How often do you self-medicate?

- Very often
- Occasionally
- Very rare
- Never

19. Which are the main reasons for self-medication?

- High costs of medical consultations
- Difficult access to a physician
- Previous experience with similar symptoms
- Recommendations from relatives or acquaintances
- Other reasons

20. Where do you obtain information about medications taken without prescription from?

- Internet
- Pharmacist
- Family/ friends
- Previous personal experience

21. If you obtain medications without prescription, the source is usually:

- Pharmacy
- Family/friends
- Remaining stock at home
- Online purchase

22. How serious do you think kidney problems caused by medications can be?

- Not serious
- Serious, but preventable
- Very rare, not worth worrying about
- I don't know

23. Have you ever experienced any renal or urinary symptoms related to the use of painkillers or antibiotics?

- Frequent nighttime urination
- Foamy or bloody urine
- Decreased urine output
- Swelling of ankles or face
- Back pain
- Fatigue
- Changes in urine (quantity, color, frequency)
- Headaches

V. Health-seeking behavior

24. When you experience pain, your first reaction is:

- I take a painkiller without medical recommendation
- I wait to see if it improves
- I call my family physician
- I ask the pharmacist
- I go to the emergency department

25. When you have fever or symptoms of any infection, your first reaction is:
- I take an antibiotic without prescription
- I take medication for fever
- I wait to see if it resolves
- I call my family physician
- I ask the pharmacist
- I go to emergency department

26. If your physician recommended a urine test to assess urinary tract health, you would:
- Definitely accept
- Accept only if it is free of charge
- Refuse (consider it unnecessary)
- Refuse (consider it too costly/ inconvenient)

27. The main reason you avoid a physician for medical tests is:
- Distance/ transportation difficulties
- Cost of tests/ consultations
- I believe kidneys and my body are not affected by common medications
- Lack of time

VI. Knowledge, perceptions, attitudes

28. Do you believe that medications taken without medical advice can affect the kidneys and your health overall?
- Yes
- No
- I don’t know

29. Have you ever been advised by a physician to undergo blood tests before or after NSAID or antibiotic use?
- Yes
- No
- I don’t know/ I don’t remember

30. Which sources of information do you consider most trustworthy for understanding the risks of these medications?
- Family physician
- Pharmacists
- Internet
- Public health campaigns
- Family/ acquaintances
